# Supplementary figures and images for: Quantifying cell densities and biovolumes of phytoplankton communities and functional groups using scanning flow cytometry, machine learning and unsupervised clustering
Source: PLoS One. 2018 May 10;13(5):e0196225. doi: 10.1371/journal.pone.0196225 (PMC5945019; doi:10.1371/journal.pone.0196225)

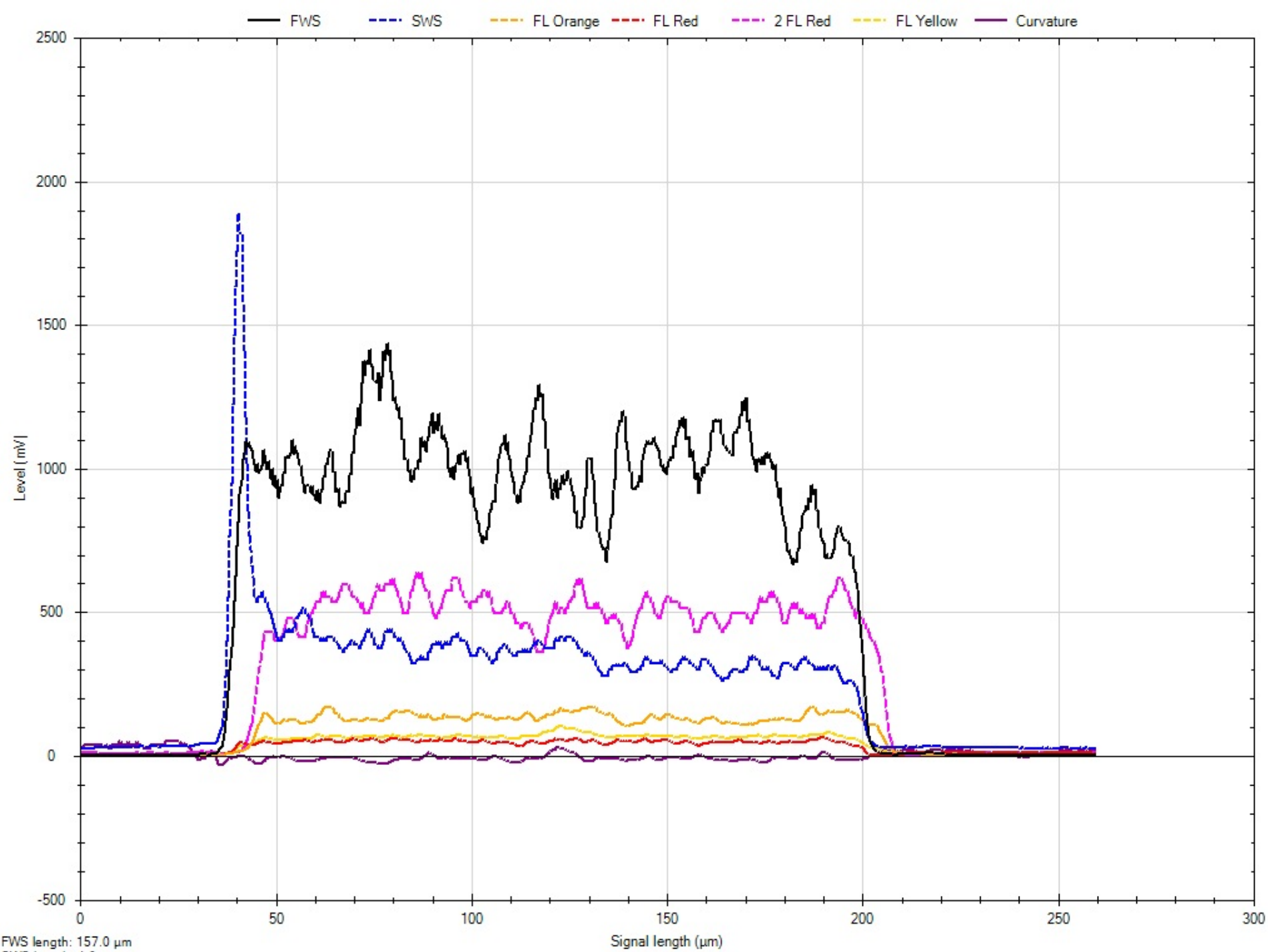

Supplement: S1 Fig — The instrument measures 6 independent pulses: Forward Scatter (FWS), Sideward Scatter (SWS), Red Fluorescence 1 (FL.Red), Red Fluorescence 2 (X2.FL.Red), Orange Fluorescence (FL.Orange) and Yellow Fluorescence (FL.Yellow). Details of the specific wavelengths of these pulses may be found in the Methods. Except for FWS, all pulses are measured by a single detector. FWS is measured by averaging the signal between 2 separate detectors. The Curvature pulse indicates the deviation between the two FWS pulses. (PDF) [file pone.0196225.s001.pdf]

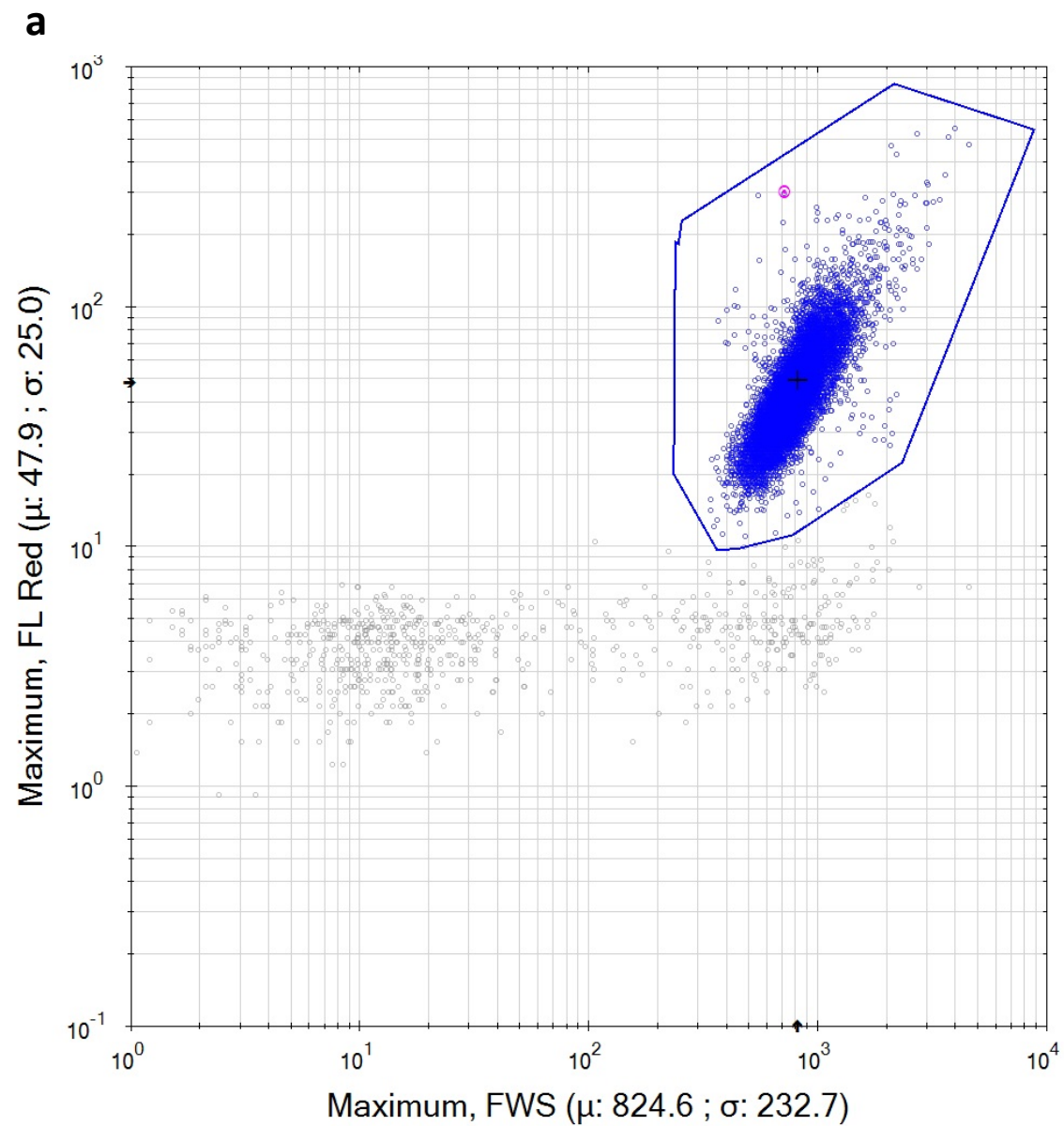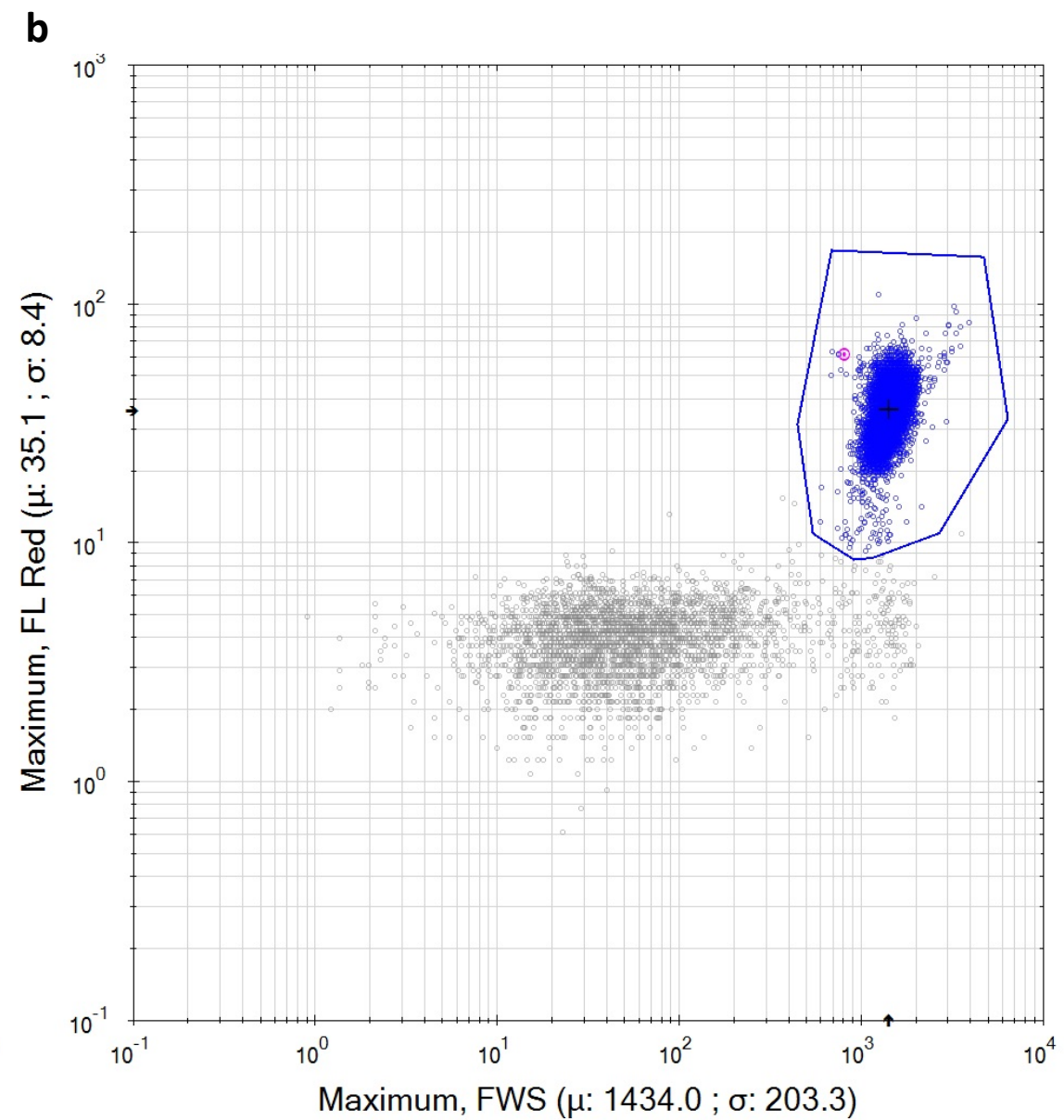

Supplement: S2 Fig — Based on prior knowledge, we manually identified the live cells and other signals in lab cultures of phytoplankton. Here we show two examples (a) Chlorella sp. and (b) Microcystis aeruginosa that illustrate this separation, using Maximum FL.Red and Maximum FWS signals. (PDF) [file pone.0196225.s002.pdf]

**a**

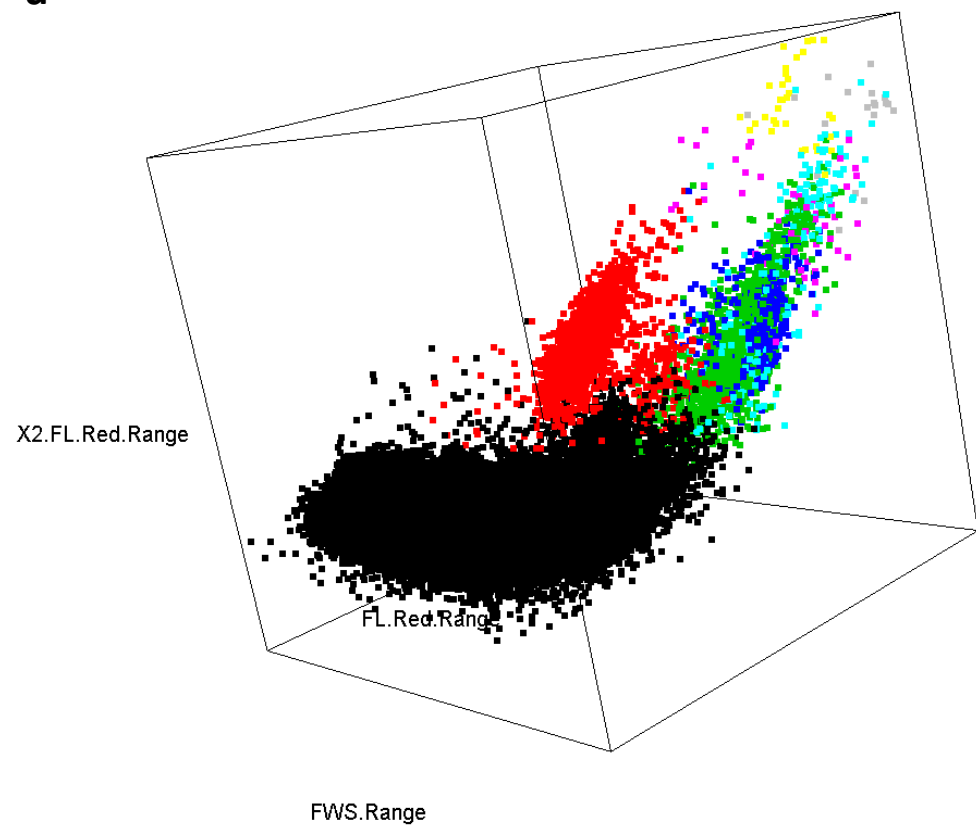

**b**

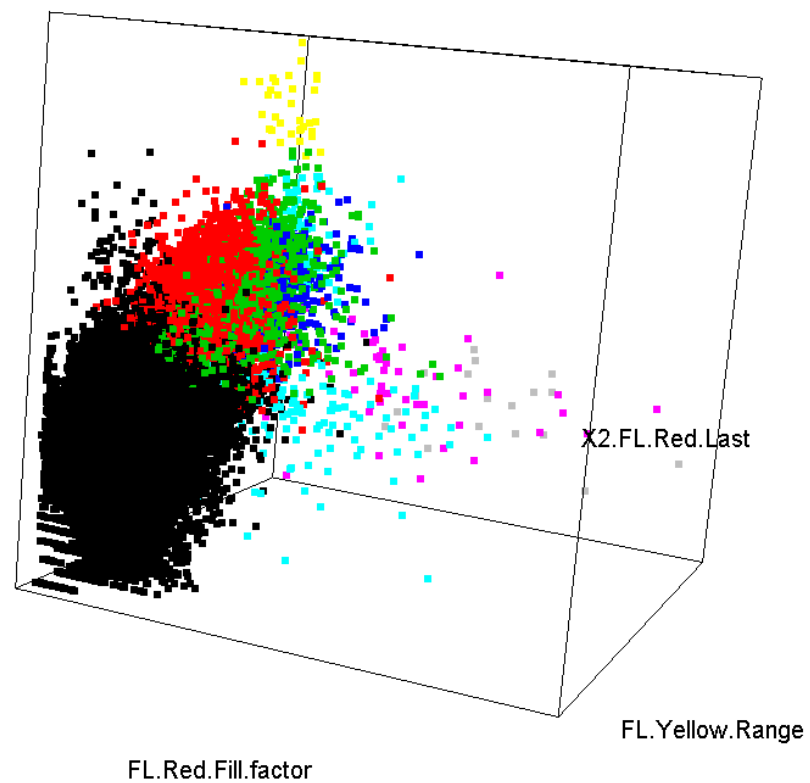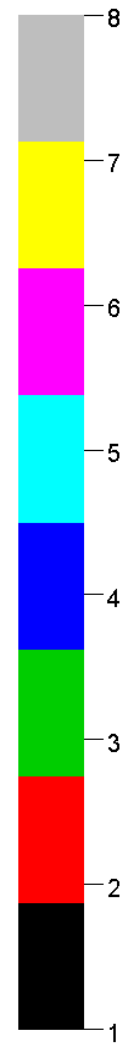

Supplement: S3 Fig — All clusters except #1 (black) were manually designated as belonging to phytoplankton cells based primarily on their high fluorescence signals. Clusters 2–8 were subsequently re-clustered (Fig 1, S4 Fig) for phytoplankton group identification, because the large proportion in cluster #1 renders the identification of smaller clusters more challenging. Axes for the plots are (a) FWS.Range, X2.FL.Red.Range & FL.Red.Range, and (b) FL.Red.Fill.factor, FL.Yellow.Range & X2.FL.Red.Last. Animated versions of these plots can be found in S1 and S2 Videos. (PDF) [file pone.0196225.s003.pdf]

**a**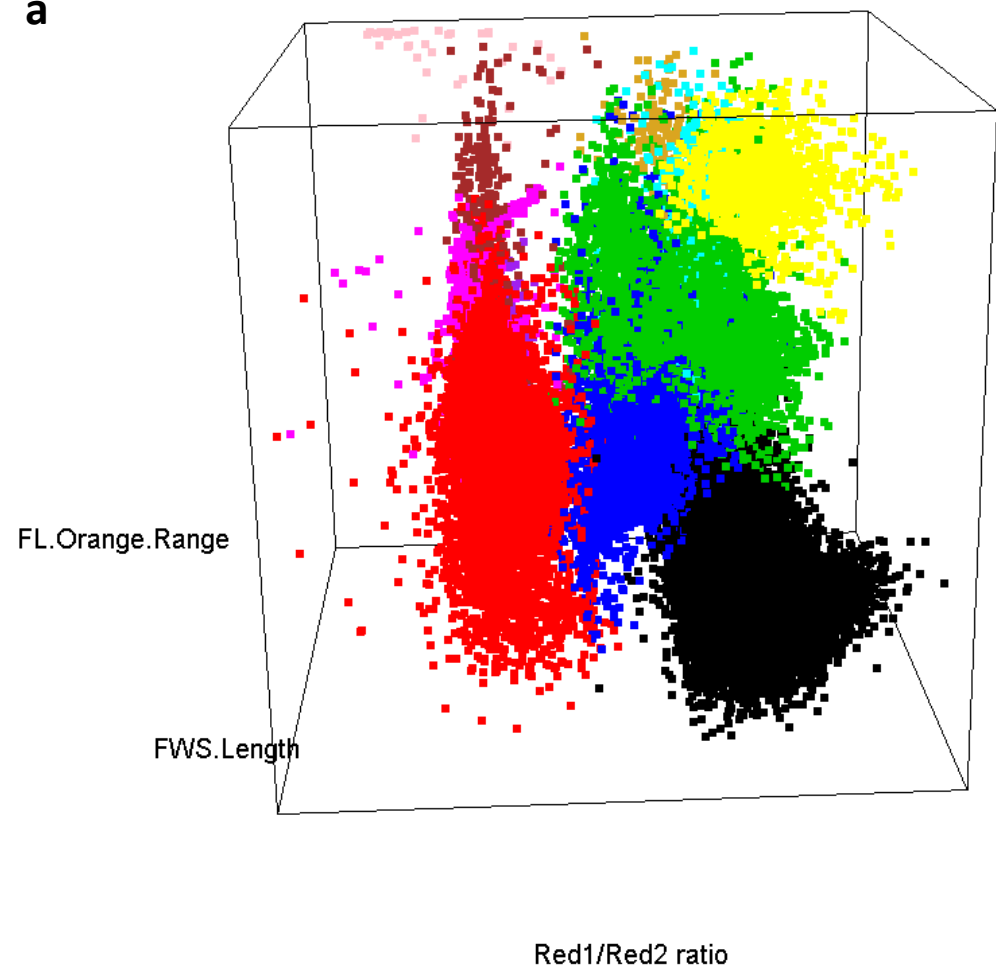**b**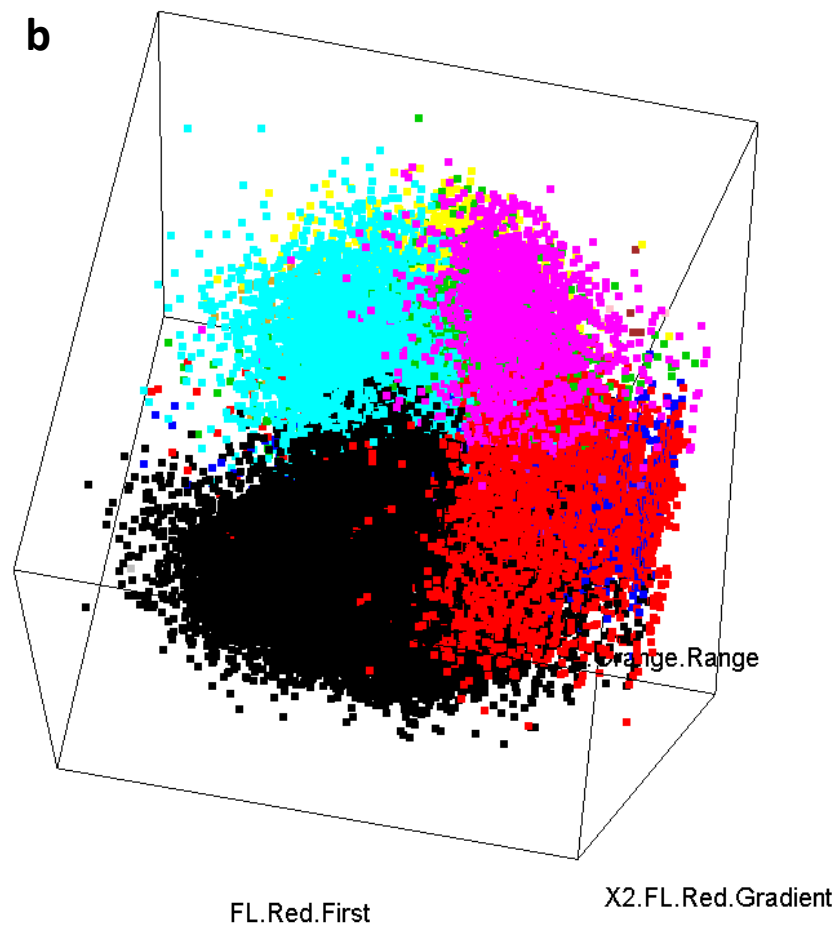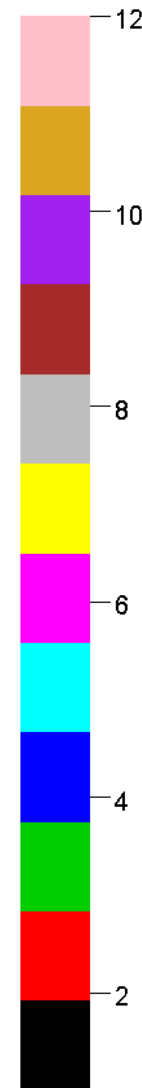

Supplement: S4 Fig — Axes for the plots are (a) Red1.Red2.ratio, FL.Orange.Range & FWS.Length, and (b) FL.Red.First, X2.FL.Red.Gradient & FL.Orange.Range. Animated versions of these plots can be found in S3 and S4 Videos. (PDF) [file pone.0196225.s004.pdf]
