# Supplementary material for: Quantifying cell densities and biovolumes of phytoplankton communities and functional groups using scanning flow cytometry, machine learning and unsupervised clustering
Source: PLoS One. 2018 May 10;13(5):e0196225. doi: 10.1371/journal.pone.0196225 (PMC5945019; doi:10.1371/journal.pone.0196225)
Supplement: S5 Fig — Importance is estimated using % change in mean squared error between trees that include individual variables and those that have those variables omitted. The 30 most important variables are shown here, but all variables were used in subsequent estimation of cell biovolumes in the field data. (PDF) [file pone.0196225.s005.pdf]

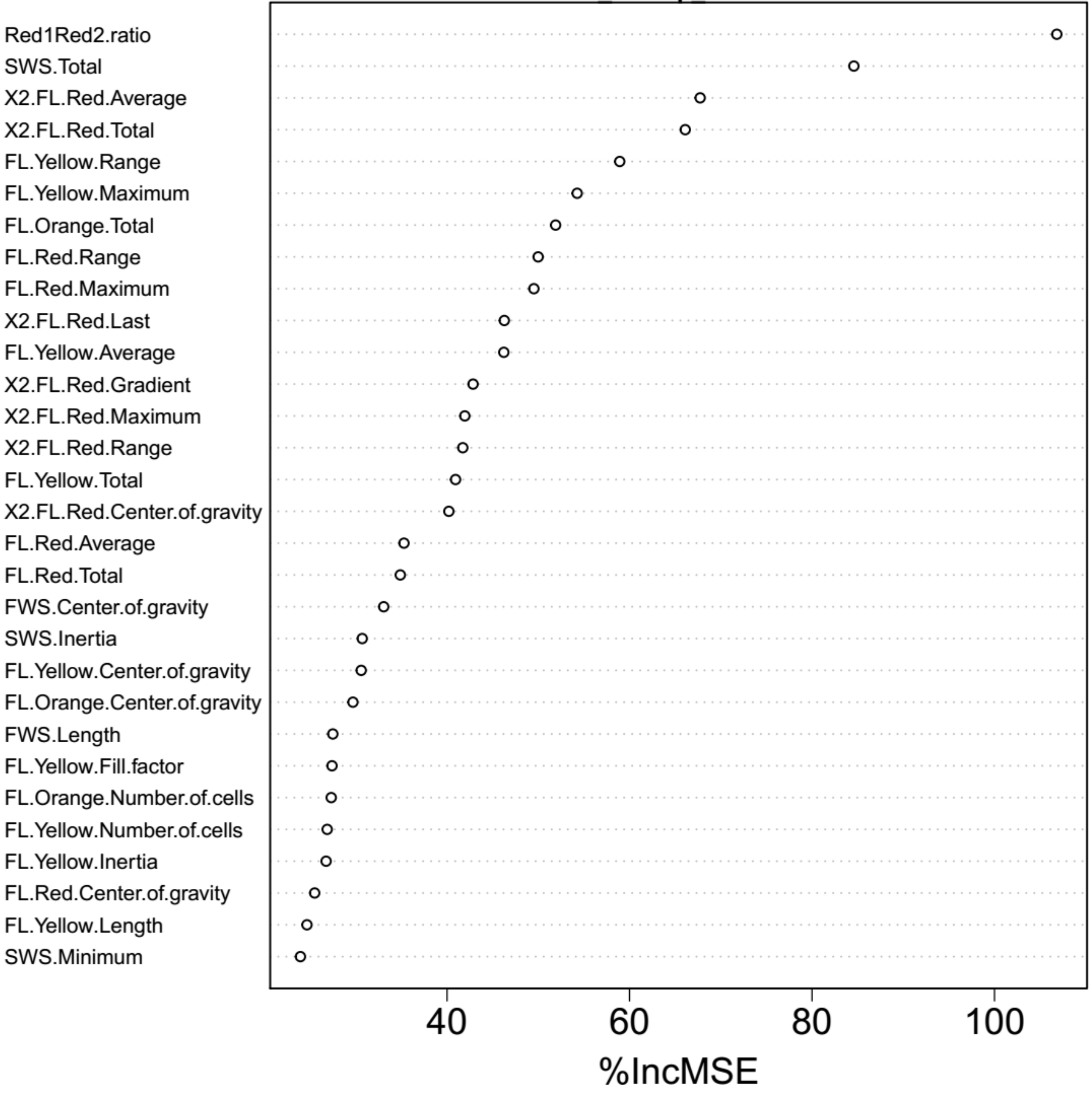
Red1Red2.ratio
SWS.Total
X2.FL.Red.Average
X2.FL.Red.Total
FL.Yellow.Range
FL.Yellow.Maximum
FL.Orange.Total
FL.Red.Range
FL.Red.Maximum
X2.FL.Red.Last
FL.Yellow.Average
X2.FL.Red.Gradient
X2.FL.Red.Maximum
X2.FL.Red.Range
FL.Yellow.Total
X2.FL.Red.Center.of.gravity
FL.Red.Average
FL.Red.Total
FWS.Center.of.gravity
SWS.Inertia
FL.Yellow.Center.of.gravity
FL.Orange.Center.of.gravity
FWS.Length
FL.Yellow.Fill.factor
FL.Orange.Number.of.cells
FL.Yellow.Number.of.cells
FL.Yellow.Inertia
FL.Red.Center.of.gravity
FL.Yellow.Length
SWS.Minimum
40
60
80
100
%IncMSE
