# Supplementary material for: Quantifying cell densities and biovolumes of phytoplankton communities and functional groups using scanning flow cytometry, machine learning and unsupervised clustering
Source: PLoS One. 2018 May 10;13(5):e0196225. doi: 10.1371/journal.pone.0196225 (PMC5945019; doi:10.1371/journal.pone.0196225)
Supplement: S1 Table — (PDF) [file pone.0196225.s006.pdf]

S1 Table. Description of CytoSense parameters

CytoSense parameter	Explanation
Total	The integrated area under each pulse
Maximum	The maximum intensity recorded for each pulse
Minimum	The minimum intensity recorded for each pulse
Range	Maximum – Minimum
Average	The average intensity of each pulse over its entire length
First	The first measured value of each pulse
Last	The last measured value of each pulse
Gradient	Absolute difference between First and Last + 0.1 (added to enable subsequent log transformation)
Length	The length of the pulse between the most extreme points that are 50% of its maximum intensity (*Full width at half maximum*), in µm
Number of cells	A continuous measure of the number of cells in a colony, generated via a Fourier transform of the pulse
Fill factor	The ratio of the area occupied by the pulse to the area of a rectangle of identical length and whose height is equal to the maximum of the pulse
Inertia	A parameter intended to capture the variability in signal height over the course of the pulse.
Center of gravity	A parameter that indicates the region of the pulse in which the signal is most concentrated.
Asymmetry	A continuous measure of the symmetry of the pulse.
Curvature	FWS is measured by averaging the signal between 2 separate detectors. The Curvature pulse indicates the deviation between the two FWS pulses.
