# Supplementary material for: Quantifying cell densities and biovolumes of phytoplankton communities and functional groups using scanning flow cytometry, machine learning and unsupervised clustering
Source: PLoS One. 2018 May 10;13(5):e0196225. doi: 10.1371/journal.pone.0196225 (PMC5945019; doi:10.1371/journal.pone.0196225)
Supplement: S2 Table — Trait values indicate the value at the centre of the clusters (PDF) [file pone.0196225.s007.pdf]

Table S2. Cluster characteristics of raw data and designated identities of the clusters based on visual inspection. Trait values indicate the value at the centre of the clusters

cluster number	proportion of particles	Designated identity	FWS. Range	FL.Red. Range	X2.FL.Red. Range	FL.Yellow. Range	X2.FL.Red. Gradient	FL.Orange. Range	FL.Red. Number. of.cells	X2.FL.Red. Last	FL.Red. Fill.factor	X2.FL.Red. Fill.factor
1	0.949	Other signals	1.550	0.530	0.730	0.006	0.117	0.549	0.446	0.504	-0.083	-0.078
2	0.022	Live cells	2.506	1.219	1.861	0.636	1.308	1.818	0.202	1.415	-0.172	-0.280
3	0.021	Live cells	3.016	2.405	1.601	0.836	1.363	0.981	0.043	1.452	-0.211	-0.269
4	0.004	Live cells	3.219	2.411	1.758	1.158	1.366	2.275	0.054	1.453	-0.254	-0.260
5	0.002	Live cells	3.320	2.677	2.051	1.311	0.865	2.056	0.141	1.151	-0.321	-0.244
6	0.001	Live cells	3.427	2.745	2.443	1.618	0.519	2.430	0.422	0.849	-0.519	-0.425
7	0.000	Live cells	3.644	2.804	3.266	1.800	2.808	2.334	0.063	2.819	-0.175	-0.218
8	0.000	Live cells	3.674	3.450	3.028	2.344	0.252	3.265	0.534	0.616	-0.693	-0.822
