# Supplementary material for: Quantifying cell densities and biovolumes of phytoplankton communities and functional groups using scanning flow cytometry, machine learning and unsupervised clustering
Source: PLoS One. 2018 May 10;13(5):e0196225. doi: 10.1371/journal.pone.0196225 (PMC5945019; doi:10.1371/journal.pone.0196225)
Supplement: S3 Table — Trait values indicate the value at the centre of the clusters. (PDF) [file pone.0196225.s008.pdf]

Table S3. Cluster characteristics of cleaned data and the designated identities for the most abundant clusters.
Trait values indicate the value at the centre of the clusters

cluster number	proportion of cells	Designated identity	FL.Red. Range	X2.FL.Red. Range	FL.Orange. Range	Red1Red2. ratio	X2.FL.Red. Gradient	FWS. Length	FWS. Fill.factor	FL.Red. First
1	0.434	Chrysophytes	2.379	1.596	0.982	0.783	1.307	0.681	-0.277	0.879
2	0.232	Cyanobacteria	1.186	1.901	1.808	-0.716	1.298	0.623	-0.370	0.252
3	0.173	Cryptophytes	2.275	1.736	2.192	0.540	1.264	0.892	-0.267	0.581
4	0.067	Greens	3.118	2.517	2.311	0.601	1.783	1.176	-0.238	1.168
5	0.046		1.620	1.428	1.606	0.192	1.081	0.812	-0.234	0.365
6	0.018		2.334	3.226	2.222	-0.892	2.737	1.240	-0.189	0.456
7	0.014		2.740	1.822	3.233	0.918	1.584	0.814	-0.301	0.741
8	0.010		2.386	1.778	1.285	0.608	0.692	1.481	-0.171	0.796
9	0.003		2.236	3.031	2.774	-0.795	0.521	1.611	-0.493	0.333
10	0.002		3.674	3.221	3.123	0.453	0.425	1.828	-0.460	1.490
11	0.002		1.566	2.256	1.916	-0.690	1.380	1.880	-0.109	0.343
12	<0.001		1.390	2.522	3.633	-1.132	0.277	2.288	-0.320	0.214
