# Supplementary material for: Quantifying cell densities and biovolumes of phytoplankton communities and functional groups using scanning flow cytometry, machine learning and unsupervised clustering
Source: PLoS One. 2018 May 10;13(5):e0196225. doi: 10.1371/journal.pone.0196225 (PMC5945019; doi:10.1371/journal.pone.0196225)
Supplement: S4 Table — (PDF) [file pone.0196225.s009.pdf]

Table S4. Characteristics of major functional groups based on lab training data

Functional group	FL.Red. Range	X2.FL.Red. Range	FL.Orange. Range	Red1Red2. ratio	X2.FL.Red. Gradient	FWS. Length	FWS.Fill. factor	FL.Red. First
Chrysophytes	1.909	1.582	1.434	0.327	0.932	0.833	-0.325	0.26
Cryptophytes	1.398	1.373	1.61	0.025	0.591	0.844	-0.31	0.243
Cyanobacteria	1.673	2.563	1.932	-0.89	1.741	1.517	-0.166	0.256
Greens	1.741	1.156	0.653	0.585	0.844	0.688	-0.226	0.404
Diatoms	2.375	1.814	1.78	0.56	0.973	1.137	-0.259	0.31
